# Automatic oxygen titration during exercise in pulmonary rehabilitation: a pilot trial

**DOI:** 10.1183/23120541.01533-2025

**Published:** 2026-07-20

**Authors:** Tessa Schneeberger, Inga Jarosch, Rainer Gloeckl, Daniela Kroll, Leonard Kolb, Wolfgang Hitzl, Andreas Rembert Koczulla

**Affiliations:** 1 Department of Pulmonary Rehabilitation, Philipps-University of Marburg, German Center for Lung Research (DZL), Marburg, Germany; 2 Institute for Pulmonary Rehabilitation Research, Schoen Klinik Berchtesgadener Land, Schoenau am Koenigssee, Germany; 3 Paracelsus Medical University, Department of Ophtalmology and Optometry, Salzburg, Austria; 4 Research Program Experimental Ophthalmology & Glaucoma Research, Paracelsus Medical University Salzburg, Salzburg, Austria; 5 Teaching hospital, Paracelsus Medical University, Salzburg, Austria

## Abstract

Dyspnoea is the predominant symptom limiting exercise tolerance in people with chronic respiratory disease [1] and is often accompanied by exertional desaturation, with or without chronic hypoxaemia, during pulmonary rehabilitation. Supplemental oxygen (O_2_) is frequently prescribed to maintain oxygen saturation measured by pulse oximetry (*S*_pO_2__) ≥90% [2, 3]. However, fixed O_2_ flow rates cannot adapt to changing O_2_ needs during activity, which may still lead to desaturation [4], increased dyspnoea and early termination of exercise, particularly during endurance training.

## Shareable abstract

**Automatic oxygen titration resulted in similar dyspnoea and physiological responses to prescribed constant-flow oxygen during exercise in pulmonary rehabilitation and was safe, suggesting potential to improve oxygen prescription in clinical practice** https://bit.ly/4bPu78W

*To the Editor:*

Dyspnoea is the predominant symptom limiting exercise tolerance in people with chronic respiratory disease [1] and is often accompanied by exertional desaturation, with or without chronic hypoxaemia, during pulmonary rehabilitation. Supplemental oxygen (O_2_) is frequently prescribed to maintain oxygen saturation measured by pulse oximetry (*S*_pO_2__) ≥90% [2, 3]. However, fixed O_2_ flow rates cannot adapt to changing O_2_ needs during activity, which may still lead to desaturation [4], increased dyspnoea and early termination of exercise, particularly during endurance training.

Automated O_2_ titration supply (ATOS) continuously adjusts O_2_ flow rates to maintain a predefined *S*_pO_2__ target (*e.g.* 92%) and have been shown to improve oxygenation and relieve dyspnoea during walking [5, 6] and daily activities [7] in patients with exertional desaturation or chronic hypoxaemia. This represents a novel approach to meeting changing needs and overcoming the limitations of fixed O_2_ flows. To date, no studies have investigated such systems during endurance exercise training, a core component of pulmonary rehabilitation programmes. This pilot trial aimed to explore dyspnoea and physiological responses to ATOS compared with constant-flow O_2_ supply (CFOS) during endurance cycle training, and to inform future trials.

This prospective, single-centre, randomised, double-blind, crossover pilot trial included two study periods: one with ATOS and one with CFOS (usual care). Each period comprised five endurance cycling sessions within 1 week. The order of O_2_ delivery modes was randomised by an independent person using a computer-generated random sequence and concealed in sequentially numbered, sealed, opaque envelopes.

Inclusion criteria were age 18–80 years and a confirmed diagnosis of chronic respiratory disease with either chronic hypoxaemia or exertional desaturation (nadir *S*_pO_2__ <88% during exercise), fulfilling established criteria for supplemental O_2_ therapy during exercise. Participants were enrolled during a 3-week inpatient pulmonary rehabilitation programme.

Exclusion criteria included an acute exacerbation of the underlying disease, unstable cardiovascular conditions, and orthopaedic or neurological limitations precluding exercise.

Before the study periods, all participants underwent a peak work rate (PWR) test on a cycle ergometer to determine individual training workload. Each study-period consisted of five 20-min supervised endurance cycling sessions performed according to current exercise recommendations for chronic respiratory diseases [8].

In the CFOS-period, O_2_ was delivered at individually prescribed flow rates for exercise, titrated during a prior exercise assessment in accordance with current O_2_ guideline recommendations [2, 3]. In the ATOS period, O_2_ flow (0–15 L·min^−1^) was automatically adjusted in real time using a closed-loop system that continuously titrated O_2_ to maintain an *S*_pO_2__ range of 90–94%, based on feedback from pulse oximetry.

A 48-h washout separated the two study periods to minimise carry-over effects. Participants, assessors and the statistician were blinded to the O_2_ delivery mode. The same device (O2matic, Herlev, Denmark) was used for both conditions, with either mode preset by an independent person. The device display was concealed to maintain blinding.

Dyspnoea, the primary outcome, was immediately assessed using the 10-point Borg scale before cycling and after cessation of cycling. Secondary physiological outcomes included continuously recorded *S*_pO_2__, transcutaneous carbon dioxide tension (*P*_tcCO_2__) and heart rate *via* the SenTec system (Switzerland), and O_2_ flow *via* the O2matic. Furthermore, leg fatigue (Borg scale), capillary blood gases (RAPIDpoint; Siemens, Germany) and inspiratory capacity (SpiroSense Pro; Pari, Germany) were obtained immediately before and after cessation of cycling.

For analysis, the mean of all endurance training sessions within each O_2_ delivery condition (CFOS and ATOS) was used to represent the respective study period. Data were checked for consistency and normality using Shapiro–Wilk test. Cross-over analysis taking the treatment and possible carry-over effects into account, and the R packages “CrossCarry” and “geepack” for cross-over designs based on generalised estimating equation models were used, and two-sided p-values <0.05 were considered statistically significant.

15 patients with chronic respiratory disease (mean±sd age: 61±9 years; 67% male; peak work rate (PWR): 51±17 W) with an indication for O_2_ therapy during exercise (duration of supplemental O_2_ use during exercise: 5.2±4.9 years) completed both study periods. Diagnoses included COPD (n=7; forced expiratory volume in 1 s (FEV_1_): 42±17% pred; total lung capacity (TLC): 119±25% pred), interstitial lung disease (n=5; forced vital capacity: 63±9% pred; TLC: 69±12% pred) and bronchiectasis (n=3; FEV_1_: 34±11% pred; TLC: 108±19% pred). Eight participants were chronically hypoxaemic (capillary oxygen tension at rest while breathing room air (*P*_cO_2rest,room-air__): 7.07±0.67 kPa) and seven had exercise-induced hypoxaemia (*P*_cO_2rest,room-air__: 8.67±0.39 kPa); all were normocapnic (capillary carbon dioxide tension at rest while breathing room air: 4.93±0.53 kPa).

Exercise intensity and workload were comparable between study periods, with participants training on average at 65±9% of PWR. Mean cycling workload was 33±11 W under CFOS and 33±10 W under ATOS (p=0.979). Mean O_2_ flow rate recorded during cycling endurance training was also similar between conditions (table 1).

**TABLE 1:** Outcomes averaged across cycle endurance training sessions during the constant-flow O_2_ supply (CFOS) and automated O_2_ titration supply (ATOS) study periods

	CFOS	ATOS	p-value
**Patients**	15	15	
**O_2_ flow rate, L·min^−1^**	3.7±1.9	3.8±2.6	0.499
**Borg scale^#^**			
Dyspnoea			
At rest	1.3±1.3	1.3±1.3	0.944
Post-exercise	4.9±1.6	4.8±1.6	0.092
Leg fatigue			
At rest	1.4±1.0	1.5±0.9	0.446
Post-exercise	4.0±1.7	3.8±1.7	0.205
**Transcutaneous measures**			
*S*_pO_2__, %	94.2±1.9	93.4±1.2	0.077
*P*_tcCO_2__, kPa	5.37±0.52	5.35±0.56	0.118
Heart rate, beats per min	99.1±12.5	93.4±1.2	0.004
**Inspiratory capacity, L**			
At rest	1.9±0.6	1.9±0.7	0.695
Post-exercise	1.7±0.7	1.7±0.7	0.286
**Blood gas analyses^¶^**			
*P*_cO_2__, kPa			
At rest	12.65±3.84	8.81±0.68	0.001
Post-exercise	8.76±1.01	9.29±1.47	0.474
*P*_cCO_2__, kPa			
At rest	4.87±0.36	4.85±0.37	0.053
Post-exercise	5.27±0.57	5.33±0.63	0.255
pH			
At rest	7.43±0.03	7.43±0.03	0.130
Post-exercise	7.38±0.05	7.38±0.05	0.563
Lactate, mmol·L^−1^			
At rest	1.5±0.5	1.5±0.3	0.862
Post-exercise	2.9±0.9	3.0±0.9	0.988

Data are present as mean±sd unless otherwise stated. Under CFOS, a fixed flow rate was applied, whereas ATOS titrated flow to maintain oxygen saturation measured by pulse oximetry (*S*_pO_2__) 92–94%. Capillary samples were obtained from the arterialised earlobe and analysed using the RAPIDPoint system (Siemens, Germany). *P*_tcCO_2__: transcutaneous carbon dioxide tension; *P*_cO_2__: capillary oxygen tension; *P*_cO_2__: capillary carbon dioxide tension. ^#^: scored from 0 to 10 points. ^¶^: blood gases measured while breathing oxygen.

Post-exercise dyspnoea ratings and baseline levels before endurance training did not differ between O_2_ delivery modes (table 1).

Leg fatigue scores, inspiratory capacity, mean *S*_pO_2__ and *P*_tcCO_2__ were also comparable between study periods. However, heart rate was statistically significant lower during the ATOS period than the CFOS period. Post-exercise blood gases also showed no significant differences (table 1).

In this pilot trial, dyspnoea did not differ between ATOS and CFOS during endurance cycle training, indicating that automated O_2_ titration provided symptom relief comparable to guideline-based exercise O_2_ titration. Importantly, ATOS achieved these comparable responses without requiring manual pre-exercise O_2_ titration. The absence of differences in dyspnoea or oxygen saturation at the end of endurance training may be explained by the use of prescribed O_2_ flow rates for exercise during CFOS, together with the use of cycling exercise, which typically induces less oxygen desaturation than walking. The slightly lower heart rate during ATOS may indicate reduced cardiovascular strain, though this should be interpreted cautiously given the small sample size.

*P*_tcCO_2__ and capillary CO_2_ levels were similar between study periods. Importantly, CO_2_ remained stable, demonstrating that automated O_2_ adjustment did not cause CO_2_ retention, confirming ATOS safety during cycling exercise, consistent with previous trials [5].

The comparable mean O_2_ flow rates between CFOS and ATOS suggest that the automated titration effectively adapted O_2_ delivery to patients’ physiological demands during cycling exercise, while achieving similar symptom responses. In routine pulmonary rehabilitation, O_2_ titration is often performed manually, which can be time-consuming and inconsistent. In many clinical settings, O_2_ flow during exercise is empirically increased by only 1 L·min^−1^ above resting flow rather than being titrated as recommended [9]. In this context, our findings indicate that average O_2_ flow automatically delivered by ATOS during exercise was retrospectively comparable to guideline-titrated O_2_ flow. This observation raises the hypothesis that an individual's mean ATOS exercise flow could inform the prescription of a personalised constant-flow for exercise. Such an approach could help standardise O_2_ titration during pulmonary rehabilitation and reduce staff workload but requires prospective validation in future studies. Finally, ATOS may have the additional advantage of avoiding unnecessary hyperoxaemia at rest compared with CFOS.

Previous studies have shown that automated-titration systems improve oxygenation and reduce dyspnoea during walking [5, 6] and daily activities [7]. The present findings extend this evidence to endurance training, which represents a core component of pulmonary rehabilitation.

This study has several limitations. The sample was small and heterogeneous, reflecting the pilot nature of the trial. The short intervention periods preclude conclusions about long-term effects.

In conclusion, ATOS during cycling endurance training achieved responses comparable to guideline-based O_2_ titration flow rates and was safe, with potential relevance for future oxygen prescription strategies.

## Data Availability

Anonymised data are available on reasonable request to the corresponding author. A data-sharing agreement will be needed.
